# Clinical Implications of Phenotypes of Hemodialysis Patients With Central Venous Occlusion or Central Venous Stenosis Defined by Cluster Analysis

**DOI:** 10.3389/fcvm.2022.901237

**Published:** 2022-06-20

**Authors:** Chunyong Wen, Bin Chen, Run Lin, Haitao Dai, Keyu Tang, Guiyuan Zhang, Jiawen Huang, Changli Liao, Linyuan Zeng, Xianhong Xiang, Jianyong Yang, Yonghui Huang

**Affiliations:** Department of Interventional Radiology, The First Affiliated Hospital, Sun Yat-sen University, Guangzhou, China

**Keywords:** central venous occlusion or stenosis, the two-step cluster analysis, primary patency, blunt stump, calcification or organization

## Abstract

**Objective:**

This study aims to investigate the association between clinical factors of patients with central (superior vena cava, brachiocephalic, or subclavian) venous occlusion or central venous stenosis (CVO/CVS) and the difficulty of interventional recanalization as well as the duration of postoperative patency.

**Methods:**

A total of 103 hemodialysis patients with CVO/CVS treated with endovascular treatment were enrolled. The two-step cluster analysis was selected to differentiate the cases into distinct phenotypes automatically. Differences in characteristics, the difficulty of interventional recanalization, and the duration of postoperative primary patency time between the two clusters were statistically compared.

**Results:**

The 103 cases were divided into distinct two clusters by the two-step cluster analysis with 48 (46.6%) in cluster 1 and 55 (53.4%) in cluster 2. Compared to cluster 2, patients in cluster 1 have a higher proportion of blunt stump, side branches, occlusion lesions >2 cm, calcification, or organization. Moreover, the above four factors were, in turn, the most critical four predictors distinguishing 103 patients into two clusters. The remaining six factors were, in turn, occlusion located in the superior vena cava (SVC), duration of central venous catheterization (CVC), lesion location, vessel diameter, number of CVC, and previously failed lesion. Of the four most important factors, with the exception of occlusion lesions exceeding 2 cm, there were significant differences in the length of procedure time between the groups grouped by the remaining three factors. And there was a significant difference in the primary patency rate between the group with blunt stump and the group without blunt stump and also between the group with occlusion lesions ≥ 2 cm and the group with occlusion lesions <2 cm. The operation time of cluster 1 was longer than that of cluster 2. In terms of postoperative patency time, the primary patency time was significantly longer in the patients of cluster 2 compared with cluster 1 (*P* = 0.025).

**Conclusion:**

Patients were divided into distinct two clusters. CVO/CVS of patients in cluster 1 was more challenging to be recanalized than that in cluster 2, and the primary patency time was significantly longer in the patients of cluster 2 compared with cluster 1. Blunt stump, side branches, occlusion lesions exceeding 2 cm, and calcification or organization are the four most critical predictors distinguishing 103 patients into two clusters.

## Introduction

Central (superior vena cava, brachiocephalic, or subclavian) venous occlusion or central venous stenosis (CVO/CVS) is one of the most frequently stated problems in hemodialysis (HD) patients with vascular access (1, 2). CVO/CVS leads to a wide range of complications, such as progressive ipsilateral arm swelling, superior vena cava syndrome, increased venous pressures, decreased access flows during hemodialysis, and prolonged bleeding after hemodialysis (3, 4). More seriously, the development of CVO/CVS eventually results in reduced long-term patency rates, which correlate with shortened survival in hemodialysis patients (5). Endovascular treatment, including percutaneous transluminal angioplasty (PTA) and stent implantation, is the preferred treatment for CVO/CVS with indications for intervention (6, 7). Many previous studies focused on postoperative patency differences between patients treated with balloon dilatation and stent implantation (8). However, a variety of clinical features affect the duration of postoperative patency and the difficulty of interventional recanalization in clinical work. Hitherto, few studies have investigated the association between clinical factors of CVO/CVS patients and the difficulty of interventional recanalization, along with the duration of postoperative patency. Stratifying patients with CVO/CVS into distinct subgroups based on clinical characteristics and then predicting the difficulty of interventional recanalization and the duration of postoperative patency of specific subgroups are of significant clinical interest to the treatment of CVO/CVS. This study aimed to apply cluster analysis to identify the different subtypes of patients with CVO/CVS and to statistically analyze the differences in the difficulty of interventional recanalization and the duration of postoperative primary patency time of distinct subgroups and eventually to explicit the clinical significance of the phenotypes defined by cluster analysis.

## Materials and Methods

### Patients and Follow-Up

This study was approved by the Ethics Committee of the First Affiliated Hospital, Sun Yat-sen University [approval number (2020)075]. Conducted as a retrospective, single-center study, a total of 103 hemodialysis patients with CVO/CVS treated with endovascular treatment were enrolled between December 2013 and December 2019. The inclusion and exclusion criteria were as follows. Inclusion criteria were as follows: (1) Patients diagnosed with CVO/CVS. (2) With indications for endovascular treatment. Exclusion criteria were as follows: (1) Patients were lacking relatively complete clinical characteristics data. (2) Patients whose procedure time was prolonged due to non-operational factors.

### Clinical Data Collection and Follow-Up

Clinical characteristics of the 103 cases are detailed in Table 1, and the primary patency time was also collected. Primary patency time was defined as the interval between the first endovascular treatment and the subsequent intervention or the first appearance of stenosis ≥50% confirmed by imaging studies. Primary patency rates were defined according to the American Association for Vascular Surgery (9). Follow-up continued until loss of follow-up, death, or the last follow-up date in December 2021.

**Table 1:** Demographic and clinical characteristics of patients in clusters.

**Variables**	***N*** **=** **103 or median** ***n*** **% or Interquartile Q 1–Q 3**	
Median age (years)	60	53–65
Gender (male/female)	53/50	51.46%/48.54%
Weight (kg)	62	52–70
Height (cm)	162	156–169
BMI	23.92	20.94–26.02
NYHA (I/II/III)	56/47	54.37%/45.63%
History of heart failure (present/absent)	11/92	10.68%/89.32%
Multivessel disease (present/absent)	34/69	33.01%/66.99%
Hypertension (present/absent)	66/37	64.07%/35.92%
Dyslipidemia (present/absent)	42/61	40.78%/59.22%
Smoking history (present/absent)	39/64	37.50%/62.50%
Diabetes (present/absent)	21/82	20.39%/79.61%
Duration of HD (months)	39	18–84
Duration of CVC (months)	8	3–36
Number of CVC	2	2–3
Previously failed lesion (present/absent)	9/94	8.74%/91.26%
Access site (fore/upper/CVC)	85/6/12	82.52%/5.83%/11.65%
Access type (AVG/AVF/CVC)	82/7/14	79.61%/6.80%/13.59%
Duration of symptom > 1 month (present/absent)	65/38	63.11%/36.89%
Lesion location (right/left/SVC)	51/32/20	49.51%/31.07%/19.42%
Occlusion site (subclavian/brachiocephalic/SVC)	28/55/20	27.18%/53.40%/ 19.42%
Side branches in 1cm (0/1/≥ 2)	51/28/24	49.51%/27.19%/23.30%
Blunt stump (present/absent)	36/67	34.95%/65.05%
Calcification or organization (present/absent)	29/74	28.16%/71.84%
Bending (present/absent)	39/64	37.86%/62.14%
Occlusion length ≥ 20 mm (present/absent)	49/54	47.57%/52.43%
Multiple lesions (present/absent)	21/82	20.39%/79.61%
Vessel diameter (mm)	13	12–15
Pro-BNP (pg/mL)	5,519	2,888–12,022
Sharp needle recanalization (present/absent)	10/93	9.7%/90.3%
Technical success (success /failure)	91/12	88.35%/11.65%
Stent implantation (present/absent)	83/20	80.58%/19.42%

*NYHA, NYHA functional status; Duration of HD, duration of hemodialysis; BMI, body mass index; CVC, central venous catheter; Previously failed lesion, lesion with a history of failed recanalization; Access site (fore/upper/CVC), the site of the dialysis access (forearm/upper arm/central venous catheter); Access type (AVG/AVF/ CVC), the type of dialysis access (arteriovenous graft/arteriovenous fistula/ central venous catheter); Lesion location (right/left/SVC), the location of the lesion (The right brachiocephalic or subclavian vein/The left brachiocephalic or subclavian vein/The superior vena cava); Occlusion site (subclavian/ brachiocephalic/SVC), the site of occlusion (The subclavian vein/The brachiocephalic vein/The superior vena cava); Side branches in 1 cm, collateral circulation within 1 cm of stenotic or occlusive lesions*.

### Endovascular Treatment

**Figure 1:**
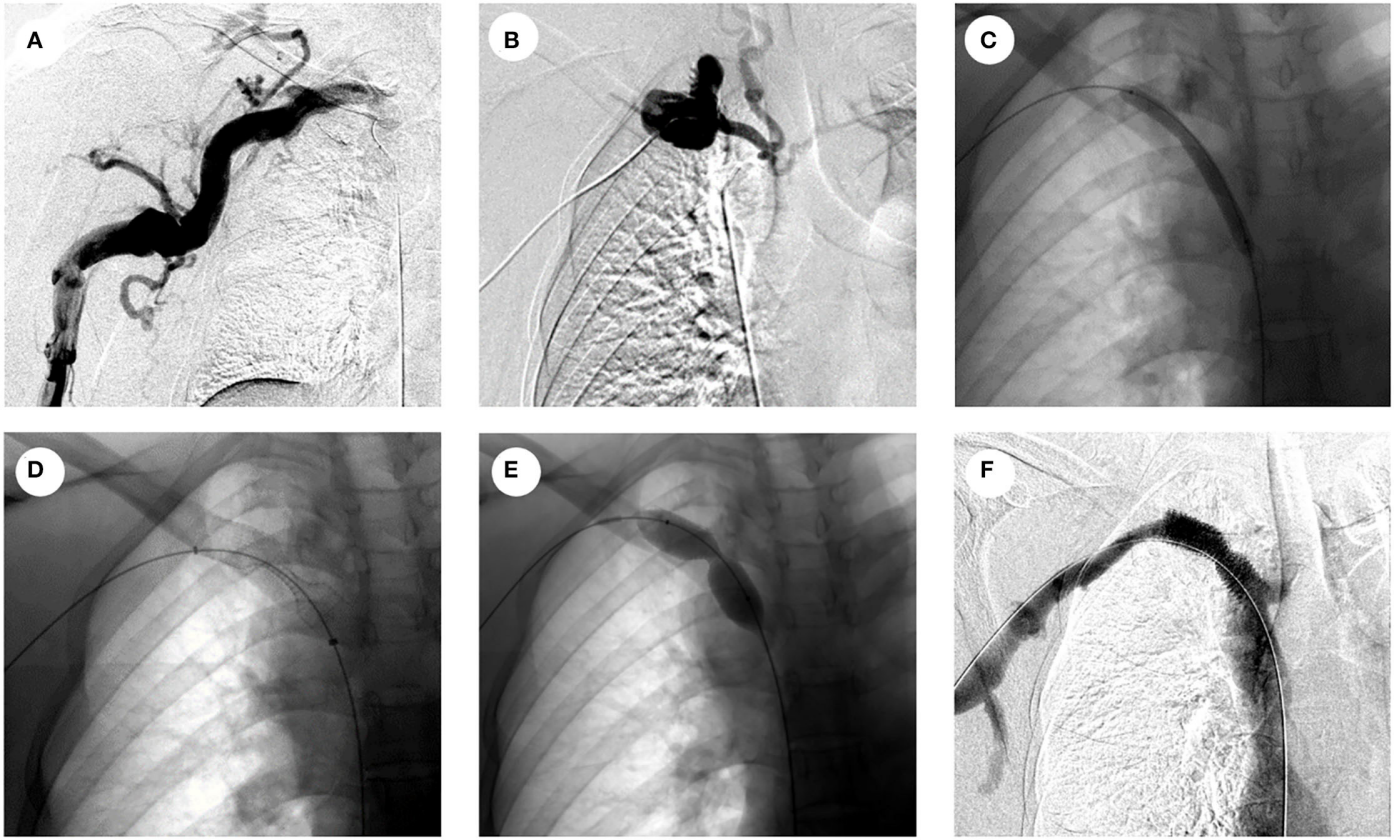
Representative pictures of sharp needle recanalization of CVO. **(A)** Venography showing occlusion in the right brachiocephalic vein; **(B)** venography showing multiple side branches around the occlusion, sharp needle recanalization was performed after the failure of the conventional method; **(C)** venography showing PTA with 6-mm balloon after sharp needle recanalization; **(D)** venography after covered stent placement; **(E)** venography showing PTA with 14-mm balloon in the stenosis of a covered stent; **(F)** venography shows significant improvement in the stenosis of the covered stent after PTA and unobstructed blood flow in the covered stent.

All the endovascular treatment of CVO/CVS was performed following current standards by interventional radiologists with abundant experience of the same team. In all cases, diagnostic venography was performed to determine the length and severity of the stenosis or occlusion. The combined femoral and jugular vein approach or combined vascular access of the upper limb and femoral vein approach was used on demand. The 0.035-inch stiff hydrophilic guidewire (Terumo Medical) was introduced to pass through the stenosis or occlusion, and then, a 4F catheter (Terumo Medical) would be placed to establish through-and-through wire access for percutaneous transluminal angioplasty (PTA) or percutaneous stent placement (PTS). Sharp recanalization with a transseptal needle would be performed when it is difficult to cross the occlusion with the conventional method, as shown in Figure 1. The specifications of the balloon or stent were determined after the multi-angle angiography. PTS was performed when residual stenosis was more than 50% after PTA. The diameters of the balloon catheters range from 10 to 18 mm, and stent diameters range from 8 to 18 mm, with lengths ranging from 40 to 60 mm. The stents used in this study include covered stents and bare stents. The covered stents used in this study were Viabahn® (W. L. Gore & Associates Inc., CA, USA), and the bare stents used were Absolute Pro (Abbott Vascular, CA, US) or Protégé™ GPS™ (ev3 Inc., MN, USA). The PTA balloon dilatation catheters used in this study were CONQUEST (Bard Peripheral Vascular Inc., AZ, USA). Venography was performed to confirm the recanalization after PAT or PTS. The procedure time of this study was defined as the time from a puncture to final recanalization or the time when the operation was terminated for some reason. Technical success was defined as successful balloon dilatation or stent placement without immediate adverse events.

### Variable Reduction by Principal Component Analysis

The variables available for this cluster analysis covering the demographic and clinical characteristics of patients are shown in Table 1. Nevertheless, not all of them could be included in the cluster analysis. Technical success, sharp needle recanalization, and stent implantation do not fall into the inherent clinical characteristics of CVO/CVS patients and therefore were not included in the cluster analysis. Besides, principal component analysis (PCA) with a varimax rotation was conducted to remove the redundancy and overlap of those features for factor extraction. Gender, height, weight, NYHA functional status, hypertension, dyslipidemia, smoking history, diabetes, access type, access site, and level of pro-BNP were excluded from the cluster analysis based on PCA results.

### Cluster Analysis

With both continuous and categorical variables in the cluster analysis, the two-step cluster analysis was selected to differentiate the cases into distinct phenotypes automatically by the intrinsic algorithm. Two-step cluster analysis allows for both the determination of cluster composition and the optimal number of clusters. Variables were included based on PCA. The clustering criterion was Schwarz's Bayesian Criterion.

### Other Statistical Analysis

Medians with IQR were performed to describe continuous variables. Differences in characteristics between the two clusters were compared. The *t*-test and Mann–Whitney U-test were used for continuous variables. The chi-square test, the Fisher exact test, and Wilcoxon rank-sum test were used for categorical variables of two clusters. The *t*-test was performed to compare the difference in the procedure time between two clusters and groups based on the four most important factors and that between the sharp needle recanalization group and the conventional method group. The primary patency time between the two clusters and groups based on the four most important factors was compared using Kaplan–Meier curves and log-rank tests. Statistical analyses were performed by SPSS version 25.0 (IBM Corp., Armonk, NY, US) and GraphPad Prism 7.0 software (GraphPad Software, USA). All of the analyses were two-sided, and *P* < 0.05 was indicated as statistical significance.

## Results

### Study Population and Follow-Up

A total of 103 cases of hemodialysis patients with CVO/CVS were enrolled. The technical success rate was 88.35%. Sharp needle recanalization was conducted in 10 out of 103 cases. A mean of 39 months of hemodialysis was observed [interquartile range (IQR) 18–84 months]. The mean duration of central venous catheterization (CVC) was 8 months (IQR 3–36 months), and the average number of CVC was 2. Nine of the 103 cases had experienced endovascular treatment failure before. In addition, 65 of the 103 cases had a duration of CVO/CVS symptoms of more than 1 month. Twenty-eight cases of the occlusion site were located in the subclavian vein, 55 cases in the brachiocephalic vein, and 20 in the superior vena cava (SVC). Twenty-four cases had no less than two side branches, 28 had one side branches, and 51 had no side branches. Blunt stump existed in 36 of the 103 cases. Calcification or organization existed in 29 of the 103 cases. Of the 103 cases, 39 had bending in the central veins with occlusion. Forty-nine of the cases had occlusion lesions exceeding 2 cm. Details of other demographic and clinical characteristics are provided in Table 1. The mean vessel diameter of CVO/CVS was 13 mm. The postoperative follow-up duration ranges from 2 to 68 months, and the average follow-up duration is 15.9 months. Restenosis occurred in 56 of 103 cases during follow-up, corresponding central veins remained patent in five patients, and 43 cases were lost to follow-up.

### Comparison of Clinical Characteristics in the Two Clusters and the Predictor Importance Chart in the Two-Cluster Model

**Figure 2:**
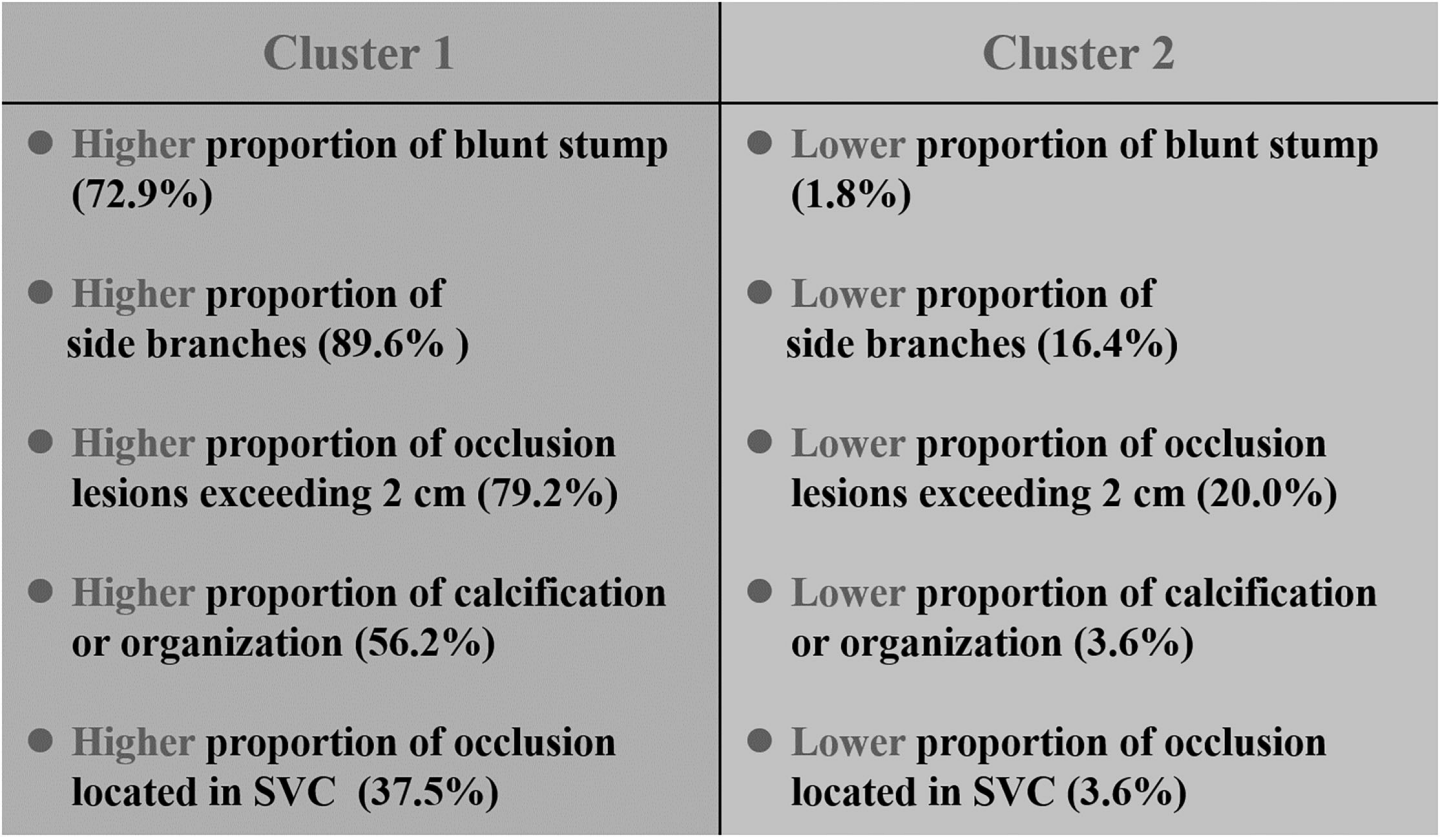
Comparison of the typical characteristics of the two clusters.

**Figure 3:**
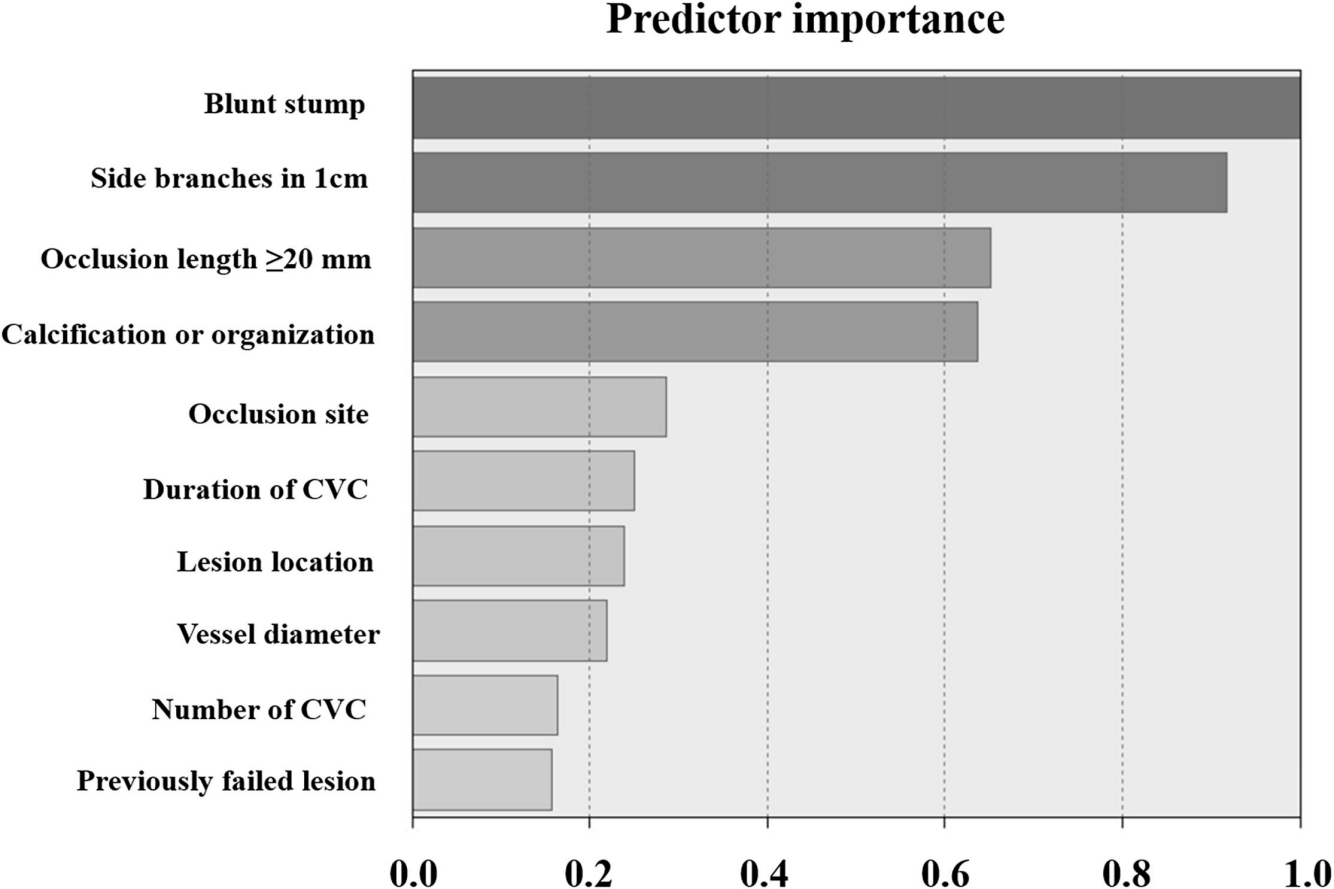
Schematic diagram of the predictor importance of various clinical characteristics in differentiating the clusters. The higher the value, the greater the importance of the factor for clustering.

**Figure 4:**
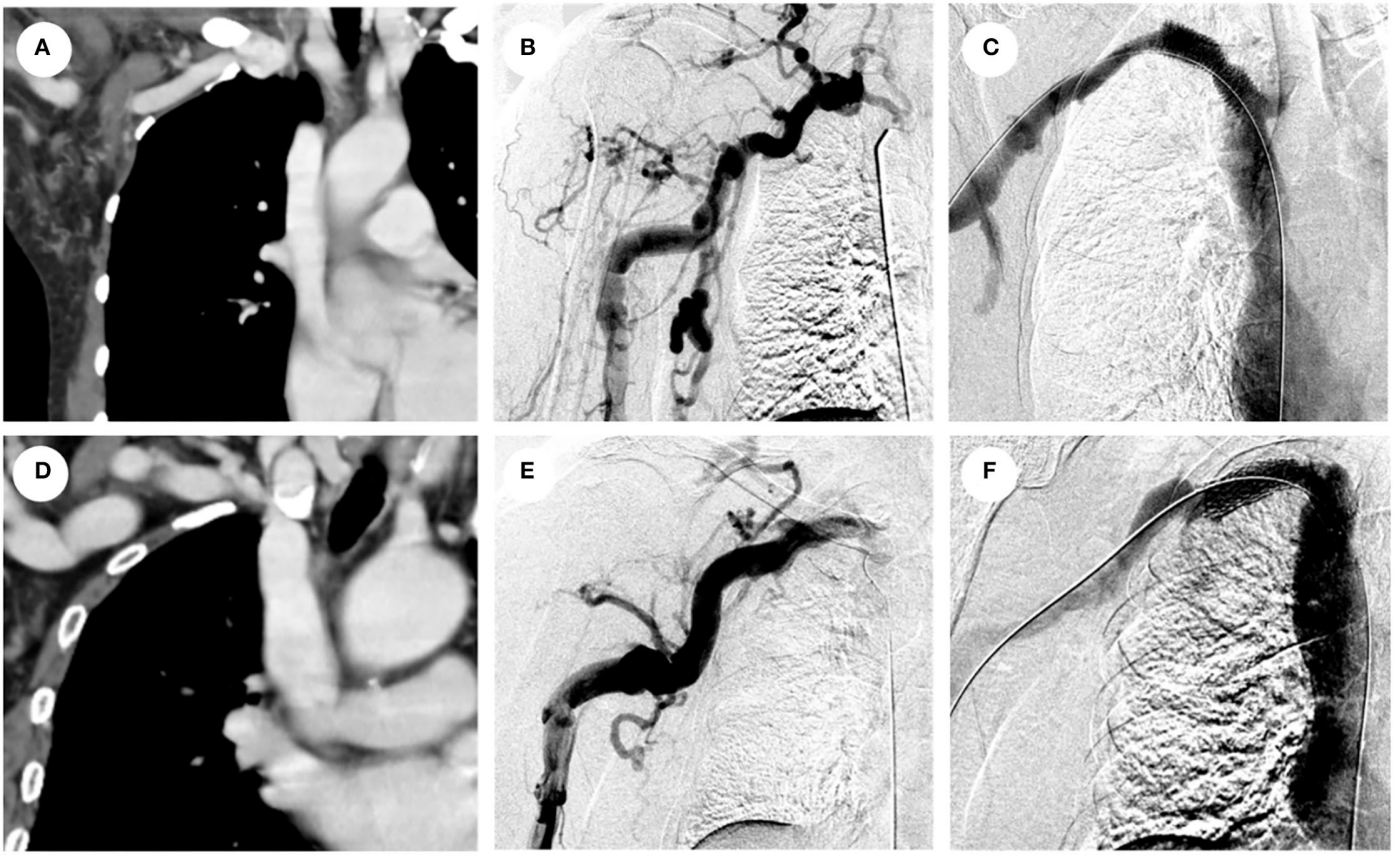
Representative images of CVO recanalization in cluster 1 and cluster 2. **(A)** Computed tomography of (CT) reconstruction of long segmental occlusion exceeding 2cm with blunt stump and calcification in the right brachiocephalic vein; **(B)** venography shows multiple collateral circulations around the occlusion; **(C)** venography showing unobstructed blood flow in the right brachiocephalic vein after covered stent placement; **(D)** CT reconstruction of stenosis <2 cm in the right subclavian vein without calcification and blunt stump; **(E)** venography showing stenosis without collateral circulation within 1 cm; **(F)** venography showing unobstructed blood flow in the right subclavian vein after covered stent placement.

One hundred and three cases were sorted into two groups (cluster 1 and cluster 2) by two-step cluster analysis. The value for the Silhouette measure of cohesion and separation in this two-cluster model is 0.2 which means the model is qualified and reliable (when the value is no <0). There are 48 cases (46.6%) in cluster 1 and 55 cases (53.4%) in cluster 2. The clinical characteristics of cluster 1 and cluster 2 are detailed in Table 2. There is a higher proportion of blunt stump, side branches, occlusion lesions exceeding 2 cm, calcification or organization, and occlusion located in SVC in cluster 1, and there are a lower proportion of side branches, occlusion lesions exceeding 2 cm, calcification or organization, and occlusion located in SVC in the cluster 2, which was summarized as typical differences between the two clusters as shown in Figure 2. The occlusion located in SVC was a factor derived from a combined analysis of the difference between the occlusion site and lesion location in the two clusters. Besides, there is a longer duration of CVC, smaller vessel diameter, more CVC in number, and more lesions with a history of failed recanalization in cluster 1 and shorter period of CVC, larger vessel diameter, less CVC in number, and fewer lesions with a history of failed recanalization in cluster 2. Except for those inherent clinical characteristics of CVO/CVS patients, a comparison of three additional factors (stent implantation, sharp needle recanalization, and technical success) was also shown in Table 2. There was no significant difference in the proportion of stent placement between the two clusters. In cluster 1, the proportion of patients treated with sharp needle recanalization was significantly higher than in cluster 2. The technical success rate of cluster 1 was lower than that in cluster 2. Figure 3 is the schematic diagram of the predictor importance of various clinical characteristics in differentiating the clusters. The results revealed that the four most important factors are blunt stump, side branches, occlusion length ≥20 mm, and calcification or organization. Other predictors and importance were detailed in Figure 3. Representative images of CVO recanalization in cluster 1 and cluster 2 are shown in Figure 4.

**Table 2:** Comparison of characteristics in two clusters.

**Variables**		**Cluster 1** ***N* = 48** **(*n* or IQR)**	**Cluster 2** ***N* = 55** **(*n* or IQR)**	***P*-value**
Blunt stump	Present	35	1	
	Absent	13	54	<0.001
Side branches in 1 cm	0	5	46	
	1	21	7	<0.001
	≥2	22	2	
Occlusion length ≥ 20 mm	Present	38	11	
	Absent	10	44	<0.001
Calcification or organization	Present	27	2	
	Absent	21	53	<0.001
Occlusion site	Subclavian	9	19	
	Brachiocephalic	21	34	<0.001
	SCV	18	2	
Duration of CVC		39.63	15.69	<0.001
Lesion location	Right	18	33	
	Left	12	20	<0.001
	SVC	18	2	
Vessel diameter		12.73	14.64	<0.001
Number of CVC		2.46	2.04	0.006
Previously failed lesion	Present	8	1	
	Absent	40	54	0.021
Duration of HD.		65.00	44.42	0.030
History of HF.	Present	7	4	
	Absent	41	51	0.231
Bending	Present	21	18	
	Absent	27	37	0.250
Multiple lesions	Present	12	9	
	Absent	36	46	0.278
Multivessel	Present	18	16	
disease	Absent	30	39	0.365
Age		59.67	57.82	0.438
Duration of symptom > 1 month	Present	29	36	
	Absent	19	19	0.597
BMI		23.63	23.64	0.983
Stent implantation	Present	35	48	
	Absent	13	7	0.066
Sharp needle recanalization	Present	9	1	
	Absent	39	54	0.010
Technical success	Success	38	53	
	Failure	10	2	0.007

### Difference in Procedure Time of Two Clusters and That of the Sharp Needle Recanalization Group and Conventional Method Group

**Figure 5:**
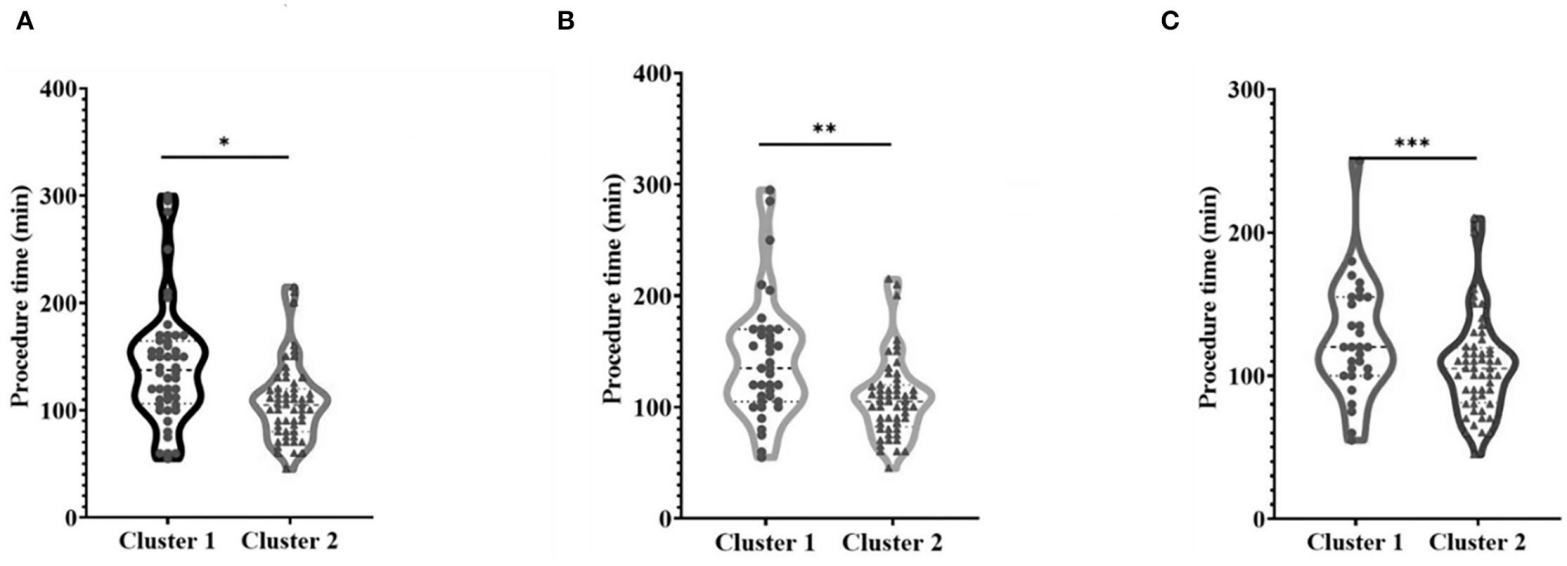
Comparison of procedure time between the two clusters. **(A)** Procedure time of the two clusters of 103 cases, *P < 0.001; **(B)** procedure time of the two clusters excluding patients with technique failure, **P = 0.001; **(C)** the procedure time of each cluster excluded patients with technique failure and those recanalized with sharp needles, ***P = 0.022.

**Figure 6:**
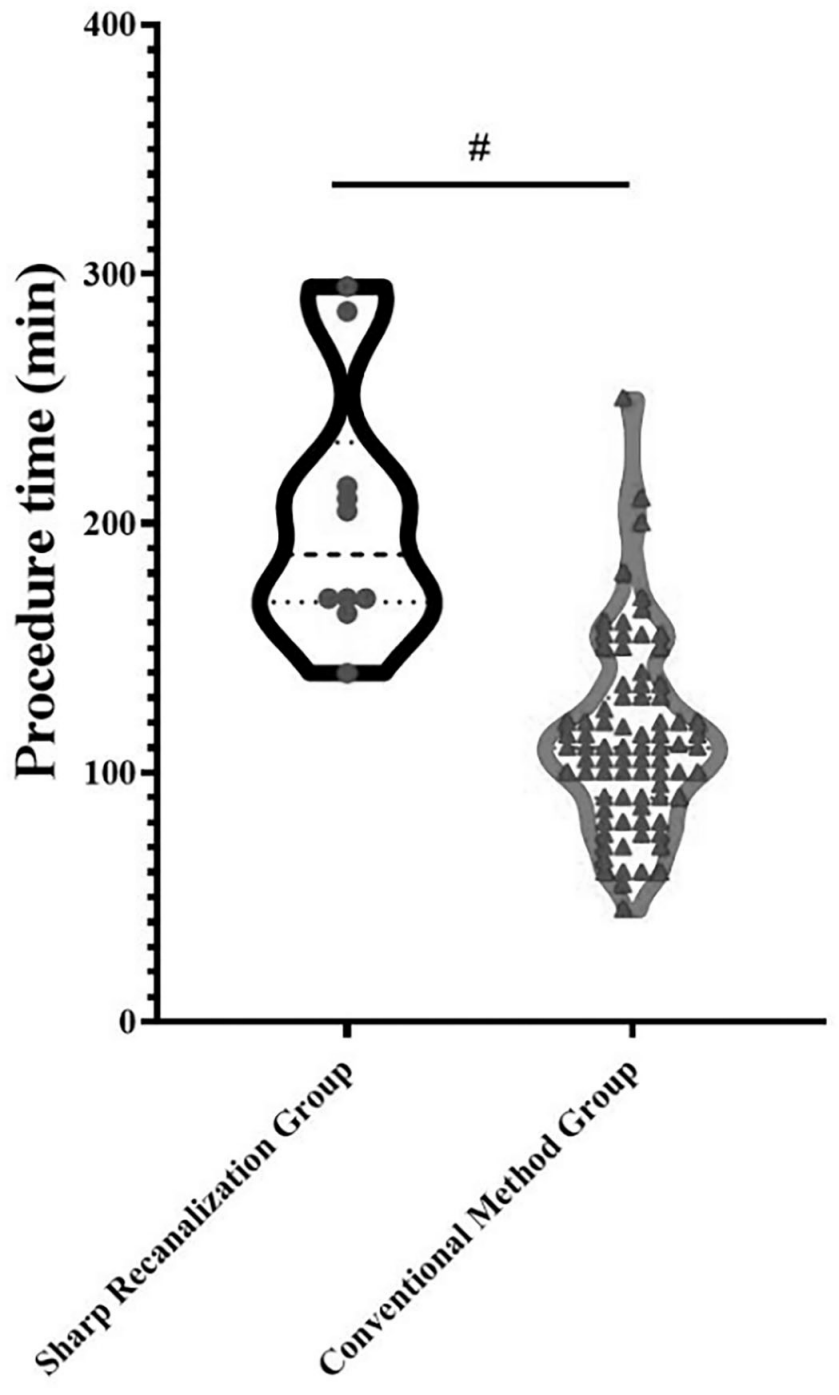
Comparison of procedure time between the sharp needle recanalization group and conventional method group; #*P* < 0.001.

Independent sample *t*-tests were performed for comparisons of procedure time between two clusters. As shown in Figure 5A, the operation time of cluster 1 was longer than that of cluster 2 (*P* < 0.001). The average operation time of cluster 1 was 142.42 min, while the mean operation duration of cluster 2 was 107.45 min. Cluster one had a significantly lower proportion of patients who achieved technical success than cluster 2. To exclude the influence of differences in the technical success rate on the results, the patients with technique failure in the two clusters were excluded from the analysis. It was shown in Figure 5B that the procedure time of patients in cluster 1 (with an average operation time of 143.00 min in 38 patients) was still significantly longer than that in cluster 2 (with an average operation time of 107.74 min in 53 patients) (*P* = 0.001) after excluding patients with technique failure. The proportion of patients treated with sharp needle recanalization in cluster 1 was significantly higher than that in cluster 2. Statistical analysis was also performed after excluding patients with technique failure and patients treated with sharp needle recanalization. As shown in Figure 5C, the procedure time of patients in cluster 1 (with an average operation time of 125.00 min in 29 patients) was significantly longer than that in cluster 2 (with an average operation time of 105.67 min in 52 patients) (*P* = 0.022). In addition, the statistical analysis was conducted in 91 cases with technical success to analyze the difference in procedure time between patients treated with sharp needle recanalization and patients treated with the conventional method. As shown in Figure 6, the operation time of the sharp needle recanalization group (with an average operation time of 202.40 min in 10 patients) was significantly longer than that of the conventional method group (with an average operation time of 112.59 min in 81 patients) (*P* < 0.001).

### Primary Patency of the Two Clusters

**Figure 7:**
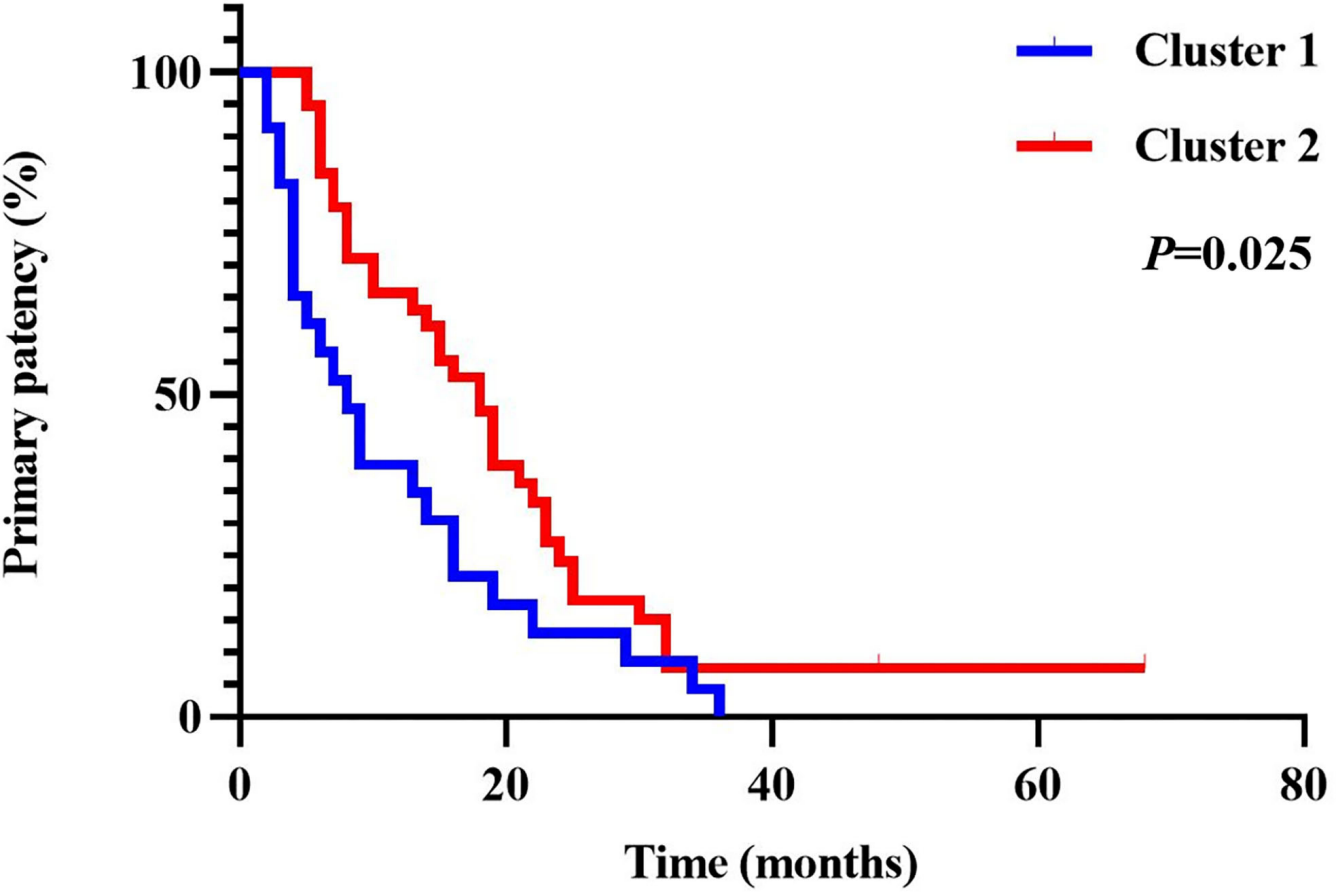
Cumulative primary patency rates between the two clusters.

The difference in the primary patency rate of the two clusters is shown in Figure 7. The figure is derived from 61 cases, 38 cases in cluster 2 and 23 cases in cluster 1. Of the 61 cases, five patients have not occurred >50% up to December 2021; all of the five patients belong to cluster 2. There were 42 patients lost to follow-up, 25 patients in cluster 1 and 17 in cluster 2. There is a significant difference in the primary patency rate between the two clusters, and patients in cluster 2 have higher 1, 2, and 3-year primary patency rates (*P* = 0.025). The primary patency was significantly longer in the patients of cluster 2 compared with cluster 1.

### Difference in Procedure Time Between Groups Based on the Four Most Important Factors

**Figure 8:**
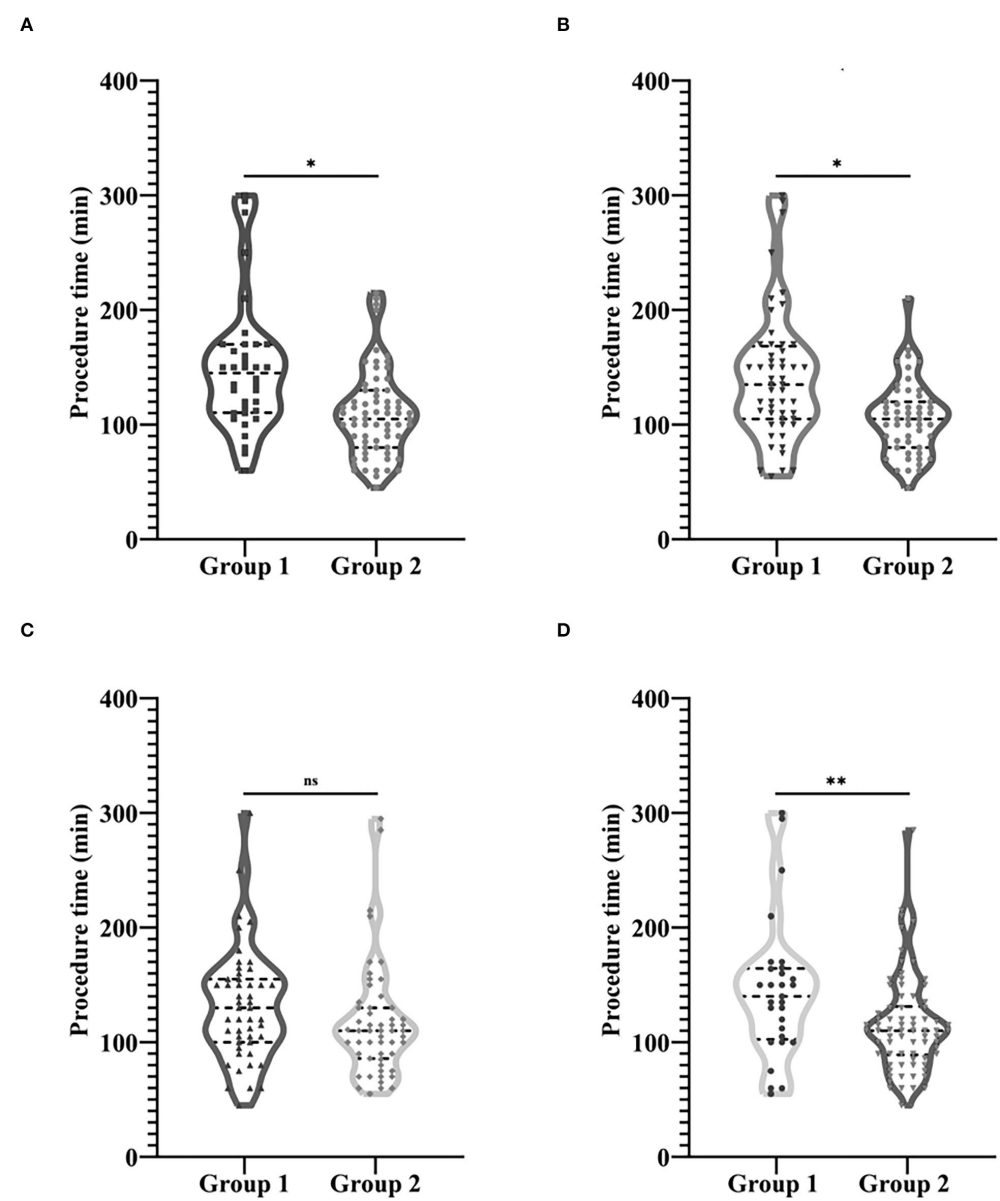
Comparison of procedure time between groups based on the four most important factors. **(A)** Group 1, Group with blunt stump; Group 2, Group without blunt stump. **(B)** Group 1, Group with side branches; Group 2, Group without side branches. **(C)** Group 1, Group with occlusion lesions exceeding 2 cm; Group 2, Group with occlusion lesions no more than 2 cm. **(D)** Group 1, Group with calcification or organization; Group 2, Group without calcification or organization. *P < 0.001, **P = 0.006.

In order to clarify the impact of the four most important factors on the length of procedure time, patients were grouped according to the four most important factors. The 103 cases were, respectively, divided into the group with blunt stump and the group without blunt stump, the group with side branches and group without side branches, the group with occlusion lesions ≥2 cm and the group with occlusion lesions <2 cm, the group with calcification or organization, and the group without calcification or organization. Independent sample *t*-tests were performed for comparisons of procedure time between groups based on the four most important factors. As shown in Figure 8, with the exception of occlusion lesions exceeding 2 cm, there were significant differences in the length of procedure time between the groups grouped by the remaining three factors. The operation time of the group with blunt stump (with an average operation time of 150.03 min in 36 patients) was significantly longer than that of the group without blunt stump (with an average operation time of 109.63 min in 67 patients) (*P* < 0.001) (Figure 8A), the operation time of the group with side branches (with an average operation time of 141.56 min in 52 patients) was significantly longer than that of the group without side branches (with an average operation time of 105.59 min in 51 patients) (*P* < 0.001) (Figure 8B), and the operation time of the group with calcification or organization (with an average operation time of 144.69 min in 29 patients) was significantly longer than that of the group without calcification or organization (with an average operation time of 115.54 min in 74 patients) (*P* = 0.006) (Figure 8D), while there was no significant difference in the length of operation time between the group with occlusion lesions ≥2 cm (with an average operation time of 131.65 min in 49 patients) and the group with occlusion lesions <2 cm (with an average operation time of 116.57 min in 54 patients) (*P* = 0.122) (Figure 8C).

### Difference in Primary Patency Between Groups Based on the Four Most Important Factors

**Figure 9:**
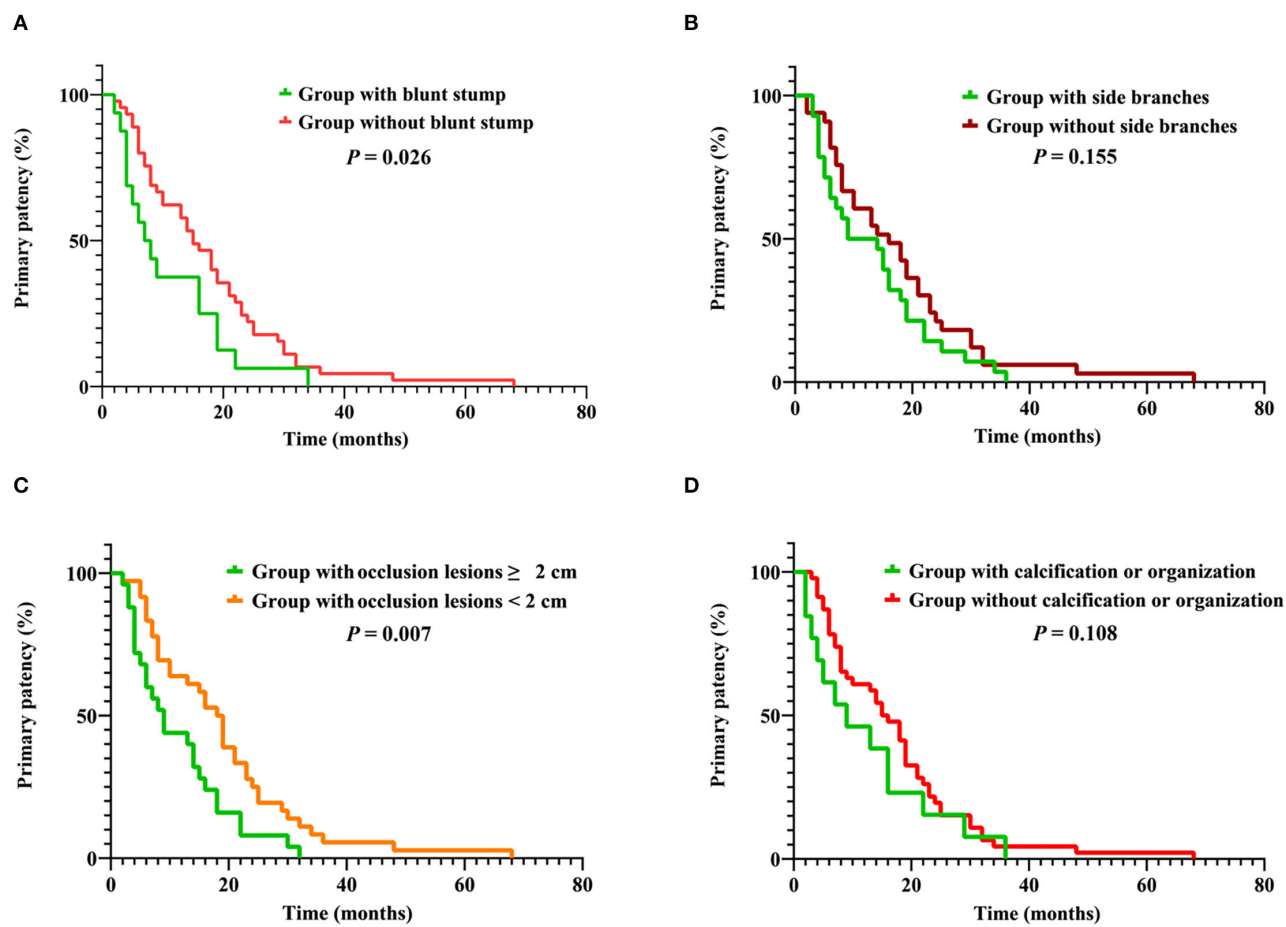
**(A)** Cumulative primary patency rates between group with blunt stump and group without blunt stump. **(B)** Cumulative primary patency rates between group with side branches and group without side branches. **(C)** Cumulative primary patency rates between group with occlusion lesions ≥ 2 cm and group with occlusion lesions <2 cm. **(D)** Cumulative primary patency rates between group with calcification or organization and group without calcification or organization.

The difference in the primary patency rate between groups based on the four most important factors was shown in Figure 9. There was a significant difference in the primary patency rate between the group with blunt stump (16 cases) and the group without blunt stump (45 cases) and also between the group with occlusion lesions ≥2 cm (25 cases) and the group with occlusion lesions <2 cm (36 cases). The primary patency was significantly longer in the patients of the group without blunt stump compared with the group with blunt stump (*P* = 0.026) (Figure 9A), and the primary patency was significantly longer in the patients of the group with occlusion lesions <2 cm compared with the group with occlusion lesions ≥2 cm (*P* = 0.007) (Figure 9C), while there was no significant difference in the primary patency between the group with side branches (28 cases) and the group without side branches (33 cases) and also between the group with calcification or organization (14 cases) and the group without calcification or organization (47 cases) (Figures 9B,D).

## Discussion

CVO/CVS is a relatively common and vital problem in HD patients, which lead to a wide range of complications and even results in decreased long-term patency rates and shortened survival (1, 3). Endovascular treatment, including PTA and stent placement, is the preferred treatment for CVO/CVS (7, 10). The decisive factors affecting the difficulty of endovascular treatment of CVO/CVS and the duration of postoperative patency are key clinical concerns. In terms of postoperative patency, multiple previous studies have compared the differences in postoperative patency rates between patients treated with PTA and PTS. Although there is no consensus on which interventional treatment has a better postoperative patency rate yet, several reports have shown no significant difference in postoperative patency rate between patients treated with stenting and PTA alone (8, 11–13). However, the relationship between the difficulty of endovascular treatment and clinical features was less mentioned in the previous literature. Different patients have unique characteristics, which influence the difficulty of endovascular treatment and postoperative patency of patients. Classifying patients based on different features and comparing the differences in duration of the procedure and postoperative patency between different clusters of patients can help to clarify the clinical significance of various clinical characteristics in patients with CVO/CVS. It is also expected to predict the duration of the interventional procedure and postoperative patency based on certain specific clinical features. In this study, patients with CVO/CVS were classified into two clusters employing two-step cluster analysis. The duration of the procedure and postoperative patency between two clusters were statistically analyzed to clarify the clinical implications of phenotypes of hemodialysis patients with CVO/ CVS.

Cluster analysis divides patients with similar clinical characteristics into the same groups, with different subgroups of patients having their specific clinicopathological characteristics so that each cluster is clinically representative. The clinicopathological characteristics data in this study were applied to the two-step cluster analysis, which divided the 103 cases into 2 clusters, 48 (46.6%) in cluster 1 and 55 (53.4%) in cluster 2. The differences in typical clinical features between patients in cluster 1 and cluster 2 are summarized in Figure 2. Compared to cluster 2, patients in cluster 1 have a higher proportion of blunt stump, side branches, occlusion lesions >2 cm, calcification, or organization. Moreover, the above four factors were, in turn, the most important four predictors distinguishing 103 patients into two clusters; the remaining six factors were, in turn, occlusion located in SVC, duration of CVC, lesion location, vessel diameter, number of CVC, and previously failed lesion. As shown in Table 2, except for these ten factors, there were also statistically significant differences in the duration of HD between the two clusters (*P* = 0.03). In addition to the inherent clinical characteristics of the patients, the differences in stent implantation, sharp needle recanalization, and technical success between the two clusters were also demonstrated in Table 2; there was no statistical difference in stent implantation between the two clusters, whereas the proportion of patients with sharp needle recanalization and the proportion of patients with technique failure were significantly higher in cluster 1 than in cluster 2.

The difference in duration of procedure time between the two clusters was statistically analyzed to compare the difficulty of endovascular treatment between the two clusters of patients. As shown in Figure 5A, the operation time of cluster 1 was longer than that of cluster 2 (*P* < 0.001). The average operation time of cluster 1 was 142.42 min, while the mean operation duration of cluster 2 was 107.45 min. Given the relatively high rate of technique failure among patients in cluster 1, excluding patients with technique failure of the two clusters was necessary for comparison. After excluding patients with technique failure, the procedure time of patients in cluster 1 (with an average operation time of 143.00 min in 38 patients) was still significantly longer than that in cluster 2 (with an average operation time of 107.74 min in 53 patients) (*P* = 0.001) as shown in Figure 5B. Similarly, the proportion of patients treated with sharp needle recanalization in cluster 1 was significantly higher than that in cluster 2. Statistical analysis was also performed after excluding patients with technique failure and patients treated with sharp needle recanalization. As shown in Figure 5C, the procedure time of patients in cluster 1 (with an average operation time of 125.00 min in 29 patients) was significantly longer than that in cluster 2 (with an average operation time of 105.67 min in 52 patients) (*P* = 0.022). In addition, to clarify the significance of sharp needle recanalization for interventional recanalization of CVO/CVS, 91 patients with successful procedures were divided into the sharp needle recanalization group and the conventional technique group, and the difference in length of procedure time between the two groups was compared. The operation time of the sharp needle recanalization group (with an average operation time of 202.40 min in 10 patients) was significantly longer than that of the conventional method group (with an average operation time of 112.59 min in 81 patients) (*P* < 0.001) as shown in Figure 6. Besides, as our team has shown in previous studies, although sharp recanalization has high technical success in the recanalization of CVO/CVS in HD patients following failure of conventional recanalization, sharp needle recanalization is commonly used for lesions that cannot be recanalized by conventional techniques (14–17). The higher proportion of patients treated with sharp needle recanalization and a higher proportion of patients with technique failure in cluster 1 also indicate that CVO/CVS of patients in cluster 1 are more difficult to recanalize than that of cluster 2. In terms of postoperative patency time, the primary patency time was significantly longer in the patients of cluster 2 compared with cluster 1 (*P* = 0.025), as was shown in Figure 7. There have been relatively few studies suggesting a difference in postoperative patency between patients with CVO/CVS treated with stent placement and PTA. Still, in our study, as seen in Table 2, there was no statistical difference in the proportion of patients treated with stent placement in cluster 1 and cluster 2. Therefore, the difference in postoperative patency between the two clusters cannot be attributed to differences in the endovascular treatment but rather to differences in the clinical characteristics of the patients themselves. In summary, 103 patients were divided into two distinct clusters by the two-step cluster analysis. CVO/CVS patients in cluster 1 were more difficult to recanalize and had shorter postoperative patency time than those in cluster 2. And compared to cluster 2, there is a higher proportion of blunt stump, side branches, occlusion lesions exceeding 2 cm, calcification or organization, and occlusion located in SVC in cluster 1. Blunt stump, side branches, occlusion lesions exceeding 2 cm, and calcification or organization are the four most important factors in differentiating the clusters.

In order to clarify the impact of the four most important factors on the length of procedure time and postoperative patency, patients were grouped according to the four most important factors. As shown in Figure 8, with the exception of occlusion lesions exceeding 2 cm, there were significant differences in the length of procedure time between the groups grouped by the remaining three factors. In terms of postoperative patency, there was a significant difference in the primary patency rate between the group with blunt stump and the group without blunt stump and also between the group with occlusion lesions ≥2 cm and the group with occlusion lesions <2 cm. A previous study of Hongsakul et al. (12) on the relationship between the type of occlusion and the difficulty of CVO recanalization showed a significantly lower technique success rate for abrupt-type occlusion, which is also known as lesions with the blunt stump in this study. Keerati Hongsakul et al. believed that the tapered-type occlusion indicates a recent occlusion. It may maintain microchannels that guidewire could pass with less resistance in contrast to occlusion with the blunt stump, while the presence of occlusion with blunt stump means that the microchannels were not maintained, which makes it more difficult to cross and more likely to induce restenosis or obstruction. Therefore, as the results of this study show, there was a significant difference both in the length of procedure time and primary patency rate between the group with blunt stump and the group without blunt stump (Figures 8A, 9A). The occurrence of side branches is one of long-term CVO/CVS complications. Side branches occur compensatorily due to the occlusion or stenosis of the central vein. The number of them is thus, to some extent, indicative of the degree and duration of CVO/CVS. Therefore, as it was shown in Figure 8B, there was a significant difference in the length of procedure time between the group with side branches and the group without side branches. In addition, although there was no significant difference in postoperative patency rate between the two groups in this study, previous studies indicated a significant relationship between the odds of the occurrence of symptomatic CVO/CVS and the number of side branches (18). This study suggests that occlusion lesions >2 cm did not increase the difficulty of recanalization (Figure 8C). In terms of postoperative patency, the primary patency was significantly longer in the patients of the group with occlusion lesions <2 cm compared with the group with occlusion lesions ≥ 2 cm (Figure 9C). There are no relevant data in the endovascular treatment of CVO/CVS before, while a negative correlation between lesion length and postoperative patency has been documented in the field of arterial interventions (19, 20). Many previous studies have suggested that calcification or organization is one of the main causes of recanalization failure (13, 21–23). As it was shown in Figure 8D, there was a significant difference in the length of procedure time between the group with calcification or organization and the group without calcification or organization, while there was no significant difference in the primary patency between the two groups (Figure 9D). Studies have shown that calcification is associated with loss of postoperative patency in the field of arterial interventions (24, 25), while there are no relevant data in the endovascular treatment of CVO/CVS before.

As patients of the cluster with a higher proportion of blunt stump, side branches, occlusion lesions exceeding 2 cm, and calcification or organization, CVO/CVS of patients in cluster 1 tend to be more difficult to be recanalized, and patients tend to have shorter postoperative patency time. In addition to the four most important predictors above, other factors may also be relevant to the difficulty of intervention and postoperative patency. Previous literature also suggests that the number of CVC and duration of CVC are independent risk factors for the development of CVO/CVS; CVO/CVS with CVC develops symptoms and occurs restenosis earlier after endovascular treatment (5, 26, 27). The results of this study may also suggest that an increase in the duration and number of CVC may also increase the difficulty of interventional recanalization and reduce postoperative primary patency time to some extent.

There are some limitations to this study. First, only primary patency time was obtained from patients. The secondary patency time has not been obtained and should be collected for further analysis in subsequent follow-ups. Second, as the interventional procedures of the 103 patients were performed by the same interventional team of one center, the duration of operation time in this study is not representative of the procedure time for patients at all medical centers and is not generalizable. Third, although 103 cases are a relatively large sample size in CVO/CVS-related studies, 103 cases are still a relatively small sample size for two-step cluster analysis even though the cluster model in this study is competent. The sample size could be further expanded in later studies.

## Conclusion

In conclusion, 103 patients were divided into two distinct clusters. Blunt stump, side branches, occlusion lesions exceeding 2 cm, and calcification or organization are the most critical four predictors distinguishing 103 patients into two clusters. Of the four most important factors, with the exception of occlusion lesions exceeding 2 cm, there were significant differences in the length of procedure time between the groups grouped by the remaining three factors, and there was a significant difference in the primary patency rate between the group with blunt stump and the group without blunt stump and also between the group with occlusion lesions ≥2 cm and the group with occlusion lesions <2 cm. As patients of the cluster with a higher proportion of blunt stump, side branches, occlusion lesions exceeding 2 cm, and calcification or organization, CVO/CVS of patients in cluster 1 tend to be more challenging to be recanalized, and patients tend to have shorter postoperative patency time. Therefore, patients with blunt stump, side branches, lesions >2 cm, calcification, or organization are more likely to require recanalization with a sharp needle and should be followed up more frequently. Early intervention should be made for any restenosis found.

## Data Availability Statement

The original contributions presented in the study are included in the article/supplementary material, further inquiries can be directed to the corresponding author/s.

## Ethics Statement

The studies involving human participants were reviewed and approved by the Ethics Committee of the First Affiliated Hospital, Sun Yat-sen University. Written informed consent for participation was not required for this study in accordance with the national legislation and the institutional requirements.

## Author Contributions

YH, CW, and BC conceived and designed the study. CW, BC, GZ, JH, CL, and LZ collected clinical data and outcome data of patients. YH, CW, BC, RL, HD, and KT participated in the endovascular treatment of patients enrolled. CW, YH, and BC wrote and revised the manuscript. RL, HD, XX, and JY provided the critical revision with insightful and constructive comments to improve the manuscript. All authors contributed to the article and approved the submitted version.

## Funding

This study was supported by Guangdong Natural Science Foundation (2020A1515010920).

## Conflict of Interest

The authors declare that the research was conducted in the absence of any commercial or financial relationships that could be construed as a potential conflict of interest.

## Publisher's Note

All claims expressed in this article are solely those of the authors and do not necessarily represent those of their affiliated organizations, or those of the publisher, the editors and the reviewers. Any product that may be evaluated in this article, or claim that may be made by its manufacturer, is not guaranteed or endorsed by the publisher.
